# Profiling of Canonical and Non-Traditional Cytokine Levels in Interferon-β-Treated Relapsing–Remitting-Multiple Sclerosis Patients

**DOI:** 10.3389/fimmu.2018.01240

**Published:** 2018-06-04

**Authors:** Chiara D’Angelo, Marcella Reale, Erica Costantini, Marta Di Nicola, Italo Porfilio, Clara de Andrés, Lidia Fernández-Paredes, Silvia Sánchez-Ramón, Livia Pasquali

**Affiliations:** ^1^Department of Medical, Oral and Biotechnological Sciences, School of Medicine and Health Sciences, University “G.d’Annunzio” Chieti-Pescara, Chieti, Italy; ^2^Department of Medicine and Ageing Sciences, School of Hygiene and Preventive Medicine, University “G.d’Annunzio” Chieti-Pescara, Chieti, Italy; ^3^Department of Neurology, Hospital General Universitario Gregorio Marañón, Madrid, Spain; ^4^Department of Clinical Immunology and IdISSC, Hospital Clínico San Carlos, Madrid, Spain; ^5^Department of Immunology, Complutense University School of Medicine, Madrid, Spain; ^6^Department of Clinical and Experimental Medicine, Neurology Unit, University of Pisa, Pisa, Italy

**Keywords:** multiple sclerosis, interferon-β therapy, pro-inflammatory cytokines, adipokines, inflammasome

## Abstract

**Background:**

Multiple sclerosis (MS) is a chronic, progressive autoimmune disease of the central nervous system in which inflammation plays a key role in the induction, development, and progression. Most of the MS patients present with relapsing–remitting (RR) form, characterized by flare-ups followed by periods of recovery. Many inflammatory and anti-inflammatory cytokines have been proposed as backers in MS pathogenesis, and the balance between these differing cytokines can regulate MS severity. Interferon (IFN)-β, a current disease-modifying therapy for MS, has demonstrated beneficial effects in reducing disease severity in MS patients. However, its immunoregulatory and anti-inflammatory actions in MS are not wholly understood. The aim of the study was to define, in clinically stable patients with RR-MS, the serum concentration of several cytokines, canonical or not, and their modulation by IFN-β therapy.

**Methods:**

Relapsing–remitting-MS patients were enrolled and diagnosed according to revised Mc Donald Diagnostic Criteria. A set of cytokines [including non-canonical neurotransmitter acetylcholine (ACh) and adipokines] and B-cell differentiation molecules, as potential biomarkers, were evaluated in 30 non-treated RR-MS patients compared to 30 IFN-β-treated MS patients and 30 age, gender, and body mass index-matched healthy controls (HC).

**Results:**

Naïve MS patients showed significantly higher levels of interleukin (IL)-1β, IL-12/IL-23p40, IL-18, high-mobility group box protein-1, and IL-18 binding protein (IL-18BP) than MS-treated patients (*p* < 0.001 for all) and HC (*p* < 0.01). IFN-β therapy has significantly downmodulated IL-1β, IL-12/IL-23p40, IL-18 to normal levels (*p* < 0.001), whereas it has decreased IL-18BP (*p* < 0.001). ACh was significantly higher in the IFN-β-treated than HC and non-treated MS patients (*p* < 0.001). No significant differences were observed either in adipokines concentration or in B-cell-associated molecules among the three study groups.

**Conclusion:**

Although more experimental evidence are required, we speculate that the efficacy of treatment of MS with IFN-β is mediated, at least in part, by its ability to work on several levels to slow down the disease progression. Proposed actions include the modulation of IL-1–inflammasome axis and modulation of ACh, B-cell activating factor/a proliferation-inducing ligand system, and several adipokines.

## Introduction

Many diseases of the central nervous system (CNS) show neuroinflammation as a common feature. Inflammatory reactions are necessary steps for removing invading agents, removing damaged cells, and helping tissue repair. However, uncontrolled neuroinflammation may lead to further tissue injury resulting in neural dysfunction or loss of function. The cross talk between periphery and CNS is crucial for the development of multiple sclerosis (MS), and understanding the complex network of signals may help to clarify its pathogenesis.

Multiple sclerosis is a CNS heterogeneous disease characterized by neuroinflammatory and neurodegenerative processes linked to the activation of autoimmune T cells, macrophages, and microglia that lead to immune responses potentiated by cytokines’ storm initiated by a positive feedback loop between immune cells and pro-inflammatory cytokines. Immune responses may be propagated within the CNS by infiltrating immune cells activated in the periphery that have crossed the blood–brain barrier (BBB) by adhesion to the endothelial cells and by the cytokines secreted by immune cells that may have direct neurotoxic properties.

Inflammasome is multiprotein intracellular complex receptors and sensors of stressors, which activates highly inflammatory signaling pathways, crucial for host defense, but whose dysregulation can in turn cause autoinflammatory and autoimmune disorders (1). NOD-like receptor family, pyrin domain containing 3 (NLRP3) inflammasome is considered a key contributor to the development of neuroinflammation and may be activated by different stimuli such as microbes, aggregated and misfolded proteins, and adenosine triphosphate, driving activation of caspase-1, and processing of interleukin (IL)-1β and IL-18, which mediate subsequent cascade immune responses (2). In experimental autoimmune encephalomyelitis (EAE), NLRP3 inflammasome induces demyelination and disease progression by promoting chemotactic migration of T helper cells, and antigen-presenting cells into the CNS (3, 4).

Interleukin-1β and IL-18 drive inflammatory responses through diverse downstream signaling pathways, leading to neuronal damage. IL-1β stimulates the activation of microglia and astrocytes, driving T cells infiltration within the CNS and inducing the production of pro-inflammatory cytokines, such as IL-6 and tumor necrosis factor (TNF)-α (5). IL-18 induces the production of adhesion molecules, pro-inflammatory cytokines, and chemokines in natural killer cells, T helper 1 (Th1), and B cells (6, 7), activates signaling pathways in microglia, and increases caspase-1 expression and pro-inflammatory cytokine production (8).

The role of B cells in MS pathology other than the production of pathogenic autoantibodies (9) has been related to activation and regulation of T cells and supported by the presence of B cells in MS lesions. Recently, it was suggested that cytokine production by B cells might be an important factor in autoimmune disease pathogenesis. In fact, B cells express TNF-β and interferon (IFN)-γ that promote Th1 differentiation (10), promote macrophage activation (11), and induce also production of IL-6, which in turn triggers EAE pathogenesis through activation of Th17 cells (12).

Both T and B cells and macrophages express components of cholinergic system, known as the non-neural cholinergic system, involved in the modulation of acetylcholine (ACh) directly produced by immune system cells (13). In several autoimmune and/or inflammatory diseases, the cholinergic anti-inflammatory system plays a central role in the immunological homeostasis (14).

Acetylcholine is a classical neurotransmitter of the central and peripheral nervous systems, and non-neuronal cells, including immune cells, can also produce and release ACh (15). The non-neuronal ACh acting through autocrine and paracrine routes may modulate inflammatory responses. At present, while it is known that ACh modulates production of IL-2 in T cells and TNF-α in macrophages, little is known about the effects exerted by ACh on dendritic cells (DCs) and B cells, other than induction of Ca^2 +^ signaling in B cells (16).

Clinical, experimental, and epidemiological studies, showing that pro-inflammatory mediators maintain microenvironmental conditions that promote loss of immune self-tolerance, have suggested the involvement of adipokines that may link the immune system with metabolic status in the pathogenesis of MS (17). Contrary to the chronic inflammatory aspect of MS, it is not fully explored whether and how adipokine levels are altered in MS patients. In fact, a pro-inflammatory environment present in MS could affect adipokine levels as well as T regulatory (Treg) functions.

Interferon-β is a first-line immunomodulatory treatment in relapsing–remitting (RR)-MS and shows beneficial effects on disease progression reducing the frequency and severity of clinical exacerbations (18). Its mechanisms of action include modulatory effects on costimulatory molecules (19) and shift from Th1 to Th2 cytokine production (20–26), and other studies have suggested modulation of Th17 development (27–29).

The protective mechanisms of IFN-β are complex and have not been completely unveiled and their failure on many patients has not been explained (30, 31). In this context, we propose to shift the attention from the brain to the role of the circulating cytokines (including non-canonical neurotransmitter ACh and adipokines) and B-cell differentiation molecules in MS patients. Thus, this study was carried out to evaluate whether IFN-β treatment may alter the circulating levels of cytokines resulting by inflammasome activation, B cells activation, and specific adipokines’ levels, to denote a novel mechanism of action for this immunomodulatory agent.

## Materials and Methods

### Clinical–Demographic Details of MS Patients and Healthy Controls (HC)

The present clinical cohort is part of a large effort on investigating genetic and immunological associations in MS. For this study, 30 HC (19 women and 11 men, median age 47 years) and 60 RR-MS patients diagnosed according to the revised McDonald diagnostic criteria (32, 33) were recruited from the Neurological Clinic, Department of Clinical and Experimental Medicine, University of Pisa and Hospital Gregorio Marañón in Madrid. The ethics committee of the institutions approved the protocol, and all patients gave their written informed consent for study inclusion, and the study was conducted in accordance with the ethical guidelines of the 2013 Declaration of Helsinki. The clinical form of RR-MS was determined according to the classification of Lublin and Reingold (34).

Inclusion criteria were as follows: age ≥ 18 years old; diagnosis of RR-MS with absence of clinical relapses at least 1 month before the inclusion in the study, and ability to provide a valid written informed consent. All subjects included had negative blood tests for other autoimmune or infectious diseases.

At the time of sampling, 30 RR-MS patients (20 women and 10 men, median age 51 years) were untreated and 30 RR-MS (18 women and 12 men, median age 43 years) were on daily subcutaneous IFN-β therapy (12 million IUs) for more than 1 year at the time of inclusion in the study. All patients were between 36 and 55 years and were ambulatory. For each patient, the estimation of disease, concomitant illnesses, and family history, and a detailed neurological examination were performed to determine the patients’ level of disability according to the Expanded Disability Status Scale (EDSS) (33).

Exclusion criteria were active infection or inflammation of any kind, and current or recent treatment with corticosteroids or any other immunomodulatory therapy during the last 6 months before inclusion in the study. Patients with relapses treated with corticosteroids within 1 month preceding enrollment were also excluded from the study.

After basic anthropometric measurements such as weight, height, and calculation of body mass index (BMI), all patients and the HC were classified as normal weight (NW). HC were frequency matched for age, gender, and BMI with MS patients and selected among the general population within the same geographical areas as MS patients. Samples were collected at the same time to diminish bias associated with the circadian cycles. Demographic and clinical data of the samples from the patients with RR-MS are presented in Table 1.

**Table 1:** Demographic and clinical data of patients with RR-MS and HC.

	RR-MS		HC (*n* = 30)	*p-*Value
	IFN-β treatment (*n* = 30)	Untreated (*n* = 30)		
Age (years)	43.0 (36.0–54.8)	51.0 (39.0–59.5)	47.0 (36.0–54.0)	0.300^a^
Gender, *n* (%)				0.585^b^
Male	12 (40.0)	10 (33.3)	11 (36.7)	
Female	18 (60.0)	20 (66.7)	19 (63.3)	
EDSS score	1.5 (1.5–2.7)	2.0 (1.0–3.4)	–	0.634^c^
Disease duration (years)	6.0 (4.0–14.0)	4.0 (2.0–8.0)	–	0.071^c^
OCB	7.0 (3.0–12.7)	8.0 (3.5–11.0)	–	0.998^c^

*Data are expressed as median and IQR*.

*^a^Kruskal–Wallis H test*.

*^b^Chi-square test*.

*^c^Mann–Whitney U test*.

*EDSS, Expanded Disability Status Scale; OCB, oligoclonal bands; HC, healthy controls; RR-MS, relapsing–remitting multiple sclerosis; IFN, interferon; IQR, interquartile range*.

### Serum Samples Collection

Blood samples (3–5 mL) were collected at least 24 h after the last IFN-β injection, from peripheral veins according to the routine puncture method. Serum was collected by blood centrifugation at 2,000 x *g* for 15 min and frozen at −80°C within 30 min, until assayed.

### Determination of Cytokine Levels

Determination of serum levels of the cytokines TNF-α, IL-1β, IL-12/23p40, IL-18, IL-18 binding protein (IL-18BP), and transforming growth factor (TGF)-β1 was performed using commercial ELISA Kits (R&D Systems Quantikine TM, Minneapolis, MN, USA). The specificity and the sensitivity for the cytokines were defined according to the manufacturer’s instructions. Minimum detectable dose (MDD) was 1.6 pg/mL for TNF-α, 1 pg/mL for IL-1β, <15 pg/μL for IL-12/23p40, 12.5 pg/mL for IL-18, 2.25 pg/mL for IL-18BP, and 15.4 pg/mL for TGF-β1. Total levels of high-mobility group box protein-1 (HMGB-1) were measured in duplicate using an available commercial ELISA kit (Elabscience Biotechnology, Wuhan, PRC) according to the manufacturer’s instructions (MDD = 18.75 pg/mL). IL-37 (MDD = 10 pg/mL) was measured in duplicate using an available commercial ELISA kit (Boster Bio. Tech., Pleasanton, CA, USA). Apelin-36 (MDD = 0.01 ng/mL), retinol-binding protein 4 (RBP-4) (MDD = 0.1 ng/mL), and Visfatin-C (MDD = 0.1 ng/mL) were measured in duplicate using an available commercial ELISA kit (Phoenix Pharm., Burlingame, CA, USA). Paired samples were measured simultaneously on the same plate, and samples with coefficients of variation higher than 10% were repeated.

### Quantification of Serum B-Cell Activating Factor (BAFF), BAFF Receptor (BAFFR), and A Proliferation-Inducing Ligand (APRIL)

All ELISA reagents were provided by Boster Bio. Tech. (Pleasanton, CA, USA). MDD was 2 pg for BAFF, 10 pg/mL for APRIL, and 10 pg/mL for BAFFR. Briefly, samples were added in duplicate on precoated plates. Next, biotinylated detection antibody was added, and samples were incubated with streptavidin-conjugated horseradish peroxidase for 30 min at room temperature. The plate was read at 450 nm. Standard curve was generated using known concentrations of recombinant analyte included in each run.

### Measurement of ACh Levels

Acetylcholine was measured by commercial colorimetric/fluorimetric kit (Abcam, Cambridge, UK). The level of Ch/ACh (pmol/well) was calculated by plotting the fluorescence of each sample in relation to choline standard curve. The measurement of the fluorescence was obtained using Glomax Multi Detection System (Promega, MI, Italy) at λ Ex/Em 535/587 nm.

### Statistical Analysis

The quantitative variables were summarized as median and interquartile range (IQR) according to their distribution and qualitative variables as frequency and percentage. A Shapiro–Wilk’s test was performed to evaluate the departures from normal distribution for each variable.

Kruskal–Wallis *H* Test was performed to evaluate differences in quantitative variables among HC, treated, and untreated RR-MS patients. Mann–Whitney *U* test was performed to evaluate differences between treated and untreated RR-MS patients.

To evaluate cytokine profile changes in patients with different EDSS scores, patients were divided on bases of median EDSS score of each group (1.5 for IFN-β-treated group and 2.0 for untreated group) in two subgroups, i.e., patients with high or low EDSS score.

Cross-tabulation analyses were conducted to assess the relationship between qualitative variables. Pearson Chi-Square test was performed to assess the statistical significance of observed relationships. Non-parametric Spearman’s correlation coefficients (Rho) were calculated to evaluate the correlation between cytokines, separately in IFN-β treated or non-treated RR-MS patients.

For all analyses, a *post hoc* test, *a priori* defined, was applied with Dunn–Sidak method adjustment for multiple comparisons. All statistical tests were evaluated at an alpha level of 0.05. Statistical analysis was performed using IBM^®^ SPSS Statistics v 20.0 software (SPSS Inc., Chicago, IL, USA).

## Results

### IL-1 Family and Inflammasome-Dependent Proteins

**Figure 1:**
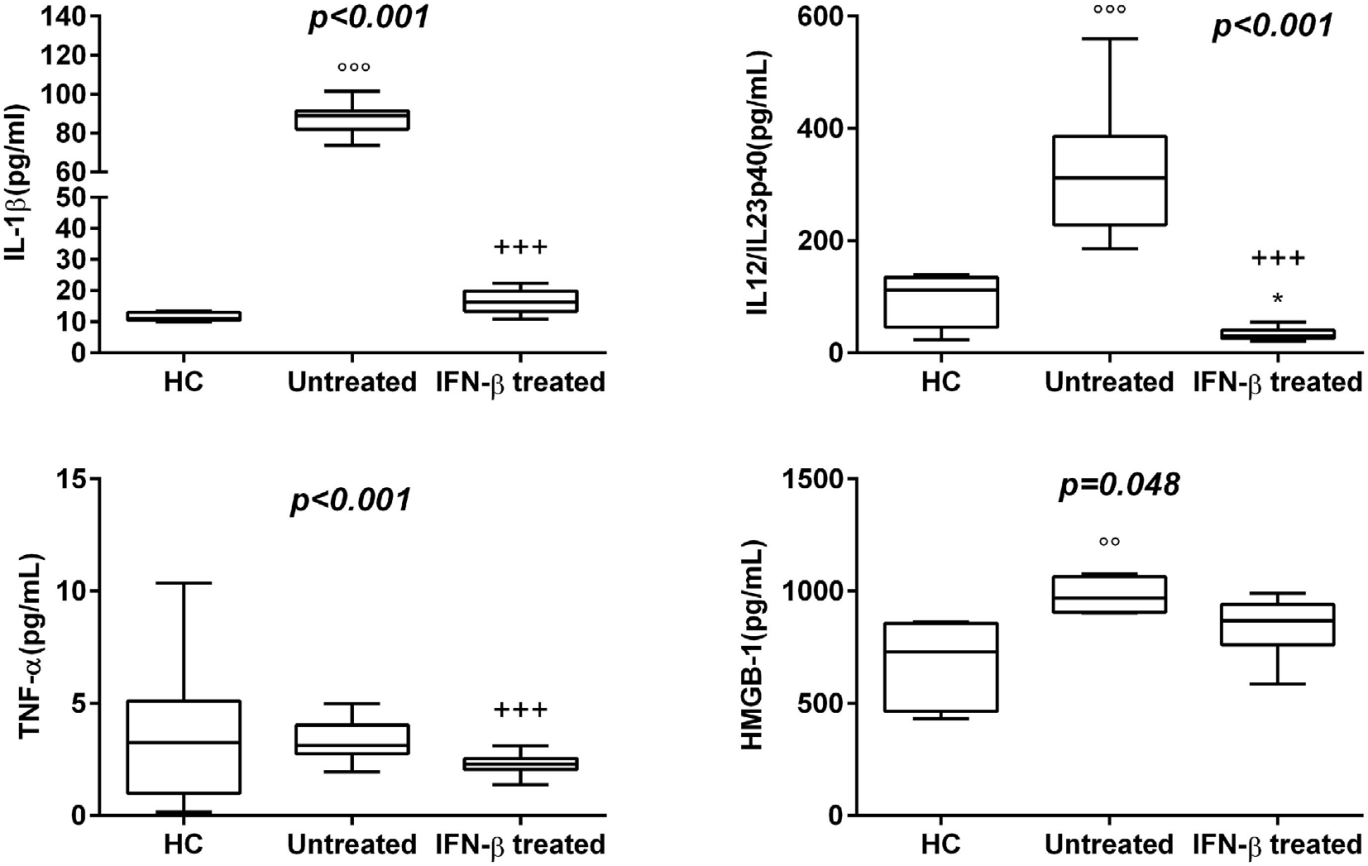
Box–whisker graphs of cytokine levels in the three study groups. Box–whisker plots show the 25th and 75th percentile range (box) with Tukey 95% confidence intervals (whiskers) and median values (transverse lines in the box). ^+++^*p* < 0.001 interferon (IFN)-β-treated vs untreated; ^ooo^*p* < 0.001, ^oo^*p* < 0.01 untreated vs healthy controls (HC); and *p < 0.05 IFN-β-treated vs HC of *post hoc* analysis. *p*-Value reported in figures are relative to Kruskal–Wallis *H* test.

**Figure 2:**
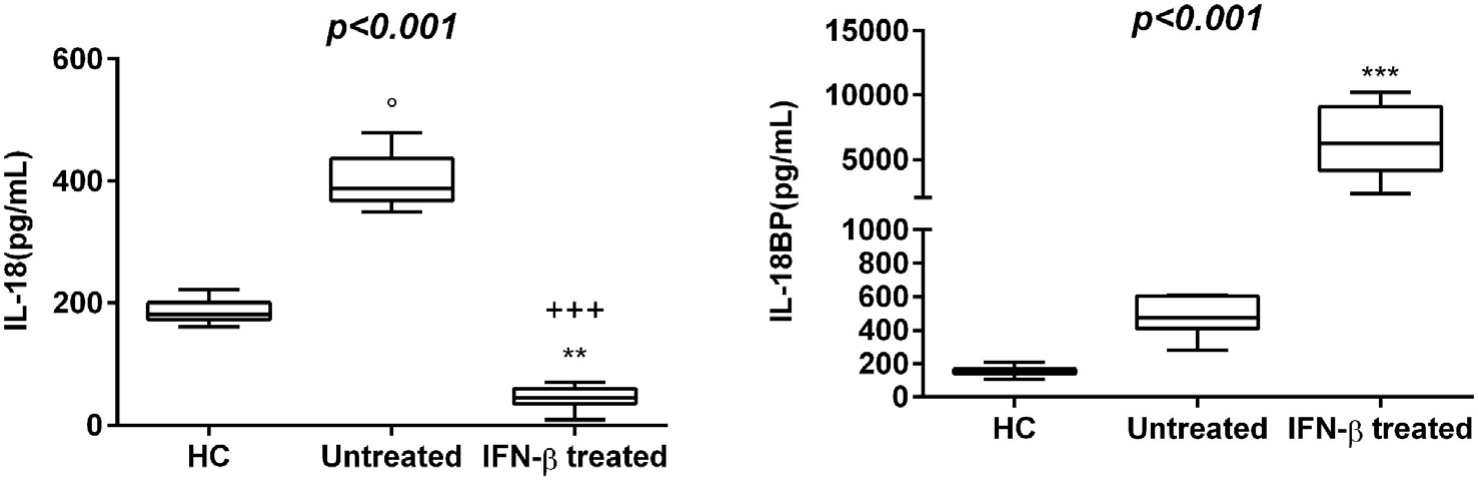
Box–whisker graphs of interleukin (IL)-18 and IL-18 binding protein (IL-18BP) levels in the three study groups. Box–whisker plots show the 25th and 75th percentile range (box) with Tukey 95% confidence intervals (whiskers) and median values (transverse lines in the box). ^+++^*p* < 0.001 interferon (IFN)-β-treated vs untreated; ^o^*p* < 0.05 untreated vs healthy controls (HC); and **p < 0.01, ***p < 0.001 IFN-β-treated vs HC of *post hoc* analysis. *p*-Value reported in figures are relative to Kruskal–Wallis *H* test.

30 newly diagnosed RR-MS subjects without medication were enrolled to evaluate the serum levels of several pro-inflammatory cytokines. The results obtained showed a statistically significant elevation of serum levels of IL-1β, IL-18, and IL-12/23p40 in untreated MS patients with respect to IFN-β treated and HC (Figures 1 and 2). Given that the activity of IL-18 is balanced by the presence of a high affinity, naturally occurring IL-18BP, a good estimation of IL-18 activity can be provided by the simultaneous detection of both IL-18 and IL-18BP. We observed an imbalance of IL-18 to IL-18BP with a higher IL-18BP/IL-18 ratio in RR-MS patients than in HC (1.2 vs 0.85, respectively). Also, the serum levels of HMGB-1 were significantly higher in the untreated RR-MS group compared to HC (31%).

Interferon-β was the most widely used therapy to treat relapsing forms of MS, albeit their specific functions are still unmet knowledge and biomarkers for therapeutic effects remain to be identified. Thus, we have analyzed which cytokines, among those increased in serum of MS patients, may be modulated by IFN-β treatment. We have found substantially lower levels of IL-1β, IL-12/23p40, TNF-α, and HMGB-1 in serum of MS patients undergoing IFN-β treatment than in serum of naïve RR-MS patients. According to these results, IFN-β-treated MS patients showed also lower IL-18 levels, while IL-18BP levels were higher than those in untreated patients (Figures 1 and 2).

Interleukin-37 is considered a natural suppressor of innate immunity included in the IL-1 family and was placed in the portfolio of classical anti-inflammatory cytokines such as TGF-β (35). In our cohort of IFN-β-treated patients, serum levels of IL-37 appeared as an increasing trend compared to untreated patients [3.38 (IQR: 2.51–4.68) vs 3.04 (IQR: 2.59–6.01)] and the same relationship was found for TGF-β1 [27,871.75 (IQR: 22,672.83−32,873.73) vs 21985.20 (IQR: 19,425.04–33,815.69)].

### Non-traditional Cytokines

In this study, levels of ACh were confirmed to be lower also in new recruited RR-MS patients without medication than in HC. Significantly elevated levels of RBP-4 were found in serum of RR-MS patients, compared to HC subjects (*p* < 0.027). Visfatin-C and Apelin-36 showed non-significantly higher levels in RR-MS patients compared to HC, but untreated RR-MS patients with EDSS < 2 showed higher levels of Visfatin.

**Figure 3:**
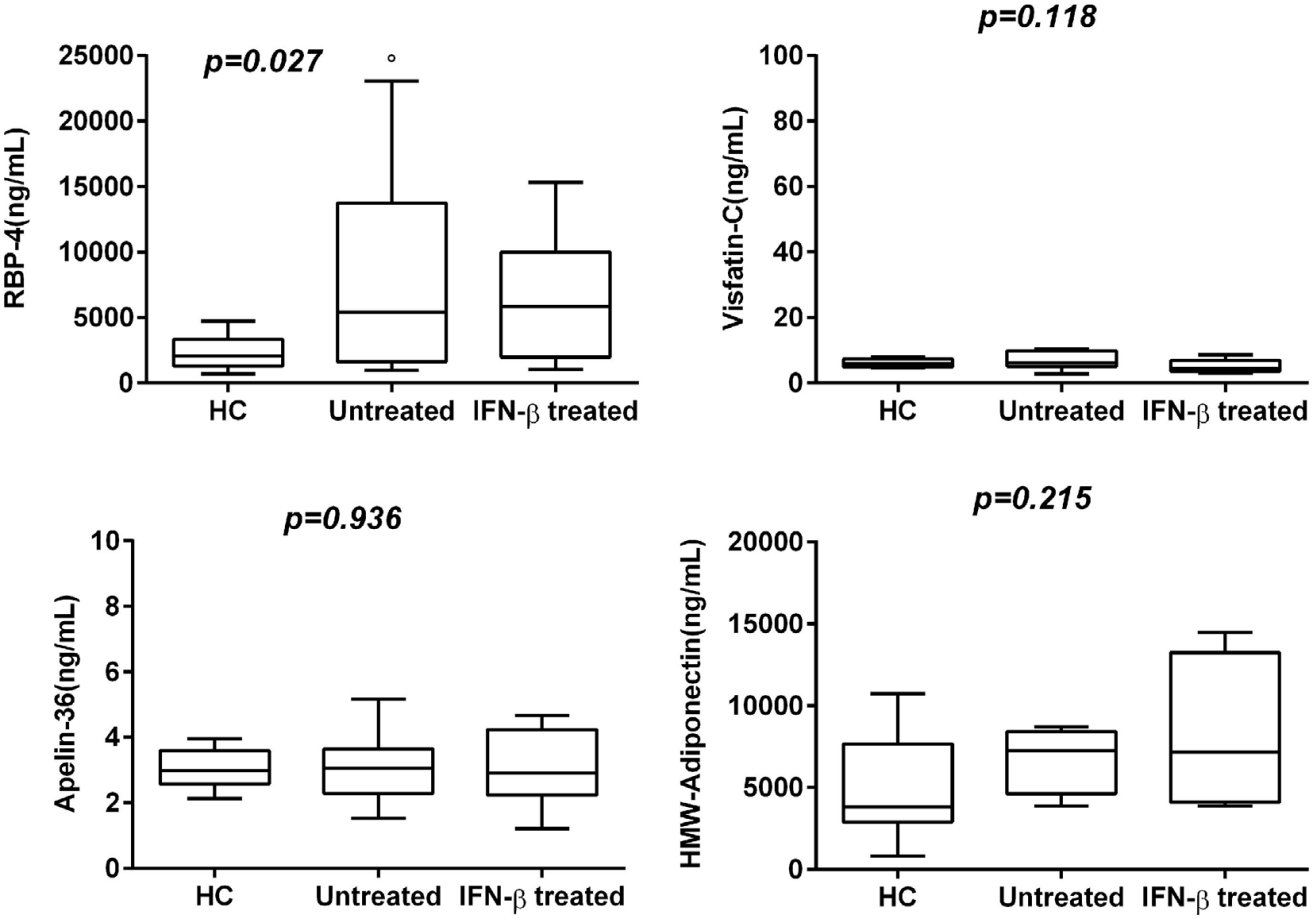
Box–whisker graphs of adipokine levels in the three study groups. Box–whisker plots show the 25th and 75th percentile range (box) with Tukey 95% confidence intervals (whiskers) and median values (transverse lines in the box). ^o^*p* < 0.05 untreated vs healthy controls (HC). *p*-Value reported in figures are relative to Kruskal–Wallis *H* test.

**Figure 4:**
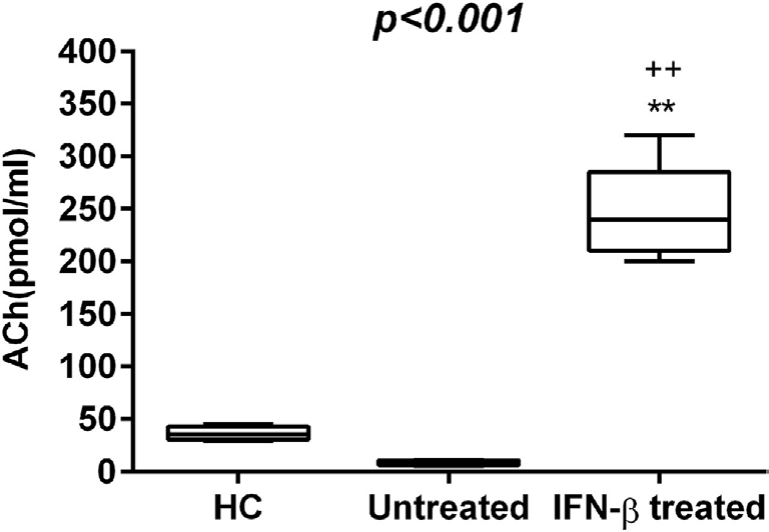
Acetylcholine (Ach) levels (pmol/mL) in the three study groups. Box–whisker plots show the 25th and 75th percentile range (box) with Tukey 95% confidence intervals (whiskers) and median values (transverse lines in the box). ^++^*p* < 0.01 IFN-β-treated vs untreated; ^**^p < 0.01 IFN-β-treated vs healthy controls (HC) *post hoc* analysis. *p*-Value reported in figures are relative to the Kruskal–Wallis *H* test.

To explore whether the IFN-β treatment influences these mediators of neural-immune and metabolic circuits, we evaluated the levels of adipokines and ACh in serum of IFN-β-treated RR-MS patients. No significant differences of Visfatin, Apelin-36, and adiponectin were detected in the IFN-β therapy group compared to the non-treated group. RBP-4 was found to be lower in the IFN-β therapy group and significantly higher in untreated RR-MS in comparison with the HC group (Figure 3). ACh levels were significantly increased under IFN-β therapy in comparison with the HC and the untreated RR-MS groups (Figure 4).

### Correlation Between Cytokines in IFN-β-Treated or Non-Treated RR-MS Patients

In our untreated patients, correlation analyses assessing associations of adipokines with IL-1β showed positive correlation with Apelin-36 levels (Rho = 0.525, *p* = 0.031) and negative correlation with Visfatin-C (Rho = −0.521, *p* = 0.050). RPB-4 negatively correlated with IL-12/23p40 (Rho = −0.700, *p* = 0.004).

On the other hand, IFN-β-treated patients showed a significant positive correlation between IL-18 and IL-18BP (Rho = 0.648, *p* = 0.043); and IL-18 and IL-12/23p40 (Rho = 0.683, *p* = 0.042).

### IFN-β Therapy and BAFF/APRIL System

The BAFF/APRIL system includes two ligands, BAFF of the TNF-family and APRIL, and the BAFFR, which is crucial for B cell homeostasis. To understand whether this system is affected by IFN-β, we have compared their serum levels in untreated and IFN-β-treated RR-MS patients (Table 2). In our IFN-β-treated RR-MS patients, we have detected a slight increase in APRIL serum levels with respect to non-treated patients (68.0 vs 50.7, *p* = 0.537). In addition, most patients under IFN-β therapy released more BAFF compared to non-treated RR-MS patients (415.6 vs 400.4, *p* = 0.662) (Table 3).

**Table 2:** Median and interquartile range of selected mediators.

	IFN-β treatment		Untreated	
	EDSS ≤ 1.5	EDSS > 1.5	EDSS ≤ 2.0	EDSS > 2.0
IL-18 (pg/mL)	46.1 (17.8; 98.1)	43.7 (38.5; 58.9)	386.6 (363.1; 447.4)	406.7 (306.2; 452.3)
IL-18BP (pg/mL)	6,710.6 (3,132.4; 9,091.3)	5,753.9 (4,310.8; 8,870.0)	500.2 (376.6; 603.5)	430.5 (354.8; 524.9)
TNF-α (pg/mL)	2.3 (2.0; 2.6)	2.2 (2.1; 2.4)	2.9 (2.4; 3.5)	3.7 (3.0; 4.3)
IL-1β (pg/mL)	14.9 (12.9; 19.4)	17.6 (15.0; 21.9)	87.3 (82.0; 91.4)	89.3 (78.0; 91.2)
IL-12/23p40 (pg/mL)	30.4 (25.5; 36.2)	38.7 (26.3; 52.3)	342.4 (251.6; 438.3)	314.8 (188.3; 404.2)
RBP-4 (ng/mL)	9,661.3 (3,033.9; 14,073.5)	4,354.5 (1,948.1; 7,696.0)	1,123.9 (944.3; 1,123.9)	3,333.5 (1,372.5; 3,333.5)
Visfatin-C (ng/mL)	4.0 (3.1; 6.8)	4.2 (4.1; 8.6)	15.8 (4.7; 15.8)	6.9 (6.3; 6.9)
Apelin-36 (ng/mL)	2.8 (2.6; 4.2)	3.7 (1.3; 5.5)	2.4 (1.8; 3.2)	3.0 (2.3; 3.0)
HMGB-1 (ng/mL)	920.5 (832.3; 978.4)	797.1 (628.9; 871.0)*	969.6 (905.9; 1,063.0)	839.5 (659.2; 930.2)°
HMW (ng/mL)	6,701.1 (3,855.6; 6,701.1)	9,626.8 (4,784.6; 9,626.8)	9,817.9 (6,590.3; 9,934.7)	7,565.7 (6,950.1; 7,565.7)
IL-37 (pg/mL)	3.4 (2.2; 3.4)	5.1 (4.1; 5.7)*	2.4 (2.2; 2.9)	4.4 (2.9; 6.2)°
TGF-β1 (pg/mL)	22,672.8 (18,058.2; 28,222.9)	29,893.6 (27,149.7; 38,249.9)*	33,891.2 (26,870.7; 38,354.9)	38,585.9 (29,054.2; 43,153.9)°
BAFFR (pg/mL)	22.5 (20.8; 22.5)	32.7 (22.4; 38.7)	23.2 (20.7; 26.2)	31.9 (27.5; 37.5)
APRIL (pg/mL)	52.2 (38.5; 52.2)	46.6 (35.2; 57.2)	43.9 (35.2; 52.3)	51.1 (38.8; 91.9)
BAFF (pg/mL)	615.1 (251.0; 615.1)	730.0 (430.0; 825.0)	87.9 (63.2; 95.8)	411.8 (366.5; 545.8)°

*p < 0.05 EDSS ≤ 1.5 vs EDSS > 1.5; °p < 0.05 EDSS ≤ 2.0 vs EDSS > 2.0 post hoc analysis.

*IFN, interferon; RR-MS, relapsing–remitting multiple sclerosis; IL, interleukin; TNF-α, tumor necrosis factor-α; TGF-β1, transforming growth factor-β1; HMGB-1, high-mobility group box protein-1; RBP-4, retinol-binding protein 4; BAFF, B-cell activating factor; BAFFR, BAFF receptor; APRIL, a proliferation-inducing ligand; IL-18BP, IL-18 binding protein*.

**Table 3:** Median and interquartile range of selected mediators.

	RR-MS		*p*-Value^a^
	IFN-β treatment (*n* = 30)	Untreated (*n* = 30)	
APRIL (pg/mL)	68.0 (44.5–100.5)	50.7 (39.4–78.4)	0.537
BAFF (pg/mL)	415.6 (230.7–792.3)	400.4 (221.6–504.9)	0.622
BAFFR (pg/mL)	27.9 (23.3–30.4)	29.8 (25.0–36.3)	0.429

*^a^p-Value relative to Mann–Whitney U test between groups*.

*IFN, interferon; APRIL, a proliferation-inducing ligand; BAFF, B-cell activating factor; BAFFR, BAFF receptor; RR-MS, relapsing–remitting multiple sclerosis*.

### Cytokine Profile Analysis Based on EDSS

Expanded Disability Status Scale was used to quantify and monitor changes in the disability in MS, by classifying MS patients into two groups: (1) representing MS patients without disability (EDSS ≤ 1.5, for IFN-β-treatment group and EDSS ≤ 2 for untreated group) and (2) including MS patients with varying degrees of disability (EDSS > 1.5 for IFN-β-treatment group and EDSS > 2 for untreated group). Since EDSS correlates with the degree of neurological dysfunction (mainly motor), potentially related to the neuroinflammation, we sought to determine whether cytokine levels vary in patients with different EDSS scores. Importantly, IL-1β, TNF-α, IL-18, IL-37, TGF-β1, RBP-4, BAFF, APRIL, and BAFFR had higher levels, while IL-18BP, IL-12/23p40, HMGB-1, and Visfatin-C had lower values in untreated patients with EDSS > 2 compared to patients with EDSS ≤ 2. In the IFN-β-treated group, IL-1β and IL-12/23p40, IL-37, TGF-β1, Apelin-36, BAFF, and BAFFR levels were higher in patients with EDSS > 1.5 than in patients with EDSS ≤ 1.5, while IL-18, IL-18BP, RBP-4, HMGB-1, and APRIL were lower in patients with EDSS > 1.5. Our results suggest that IL-1β, IL-18, IL-18BP, and IL-12/23p40 play a role in MS pathogenesis and course, since they are specifically modulated by IFN-β treatment (Table 2).

## Discussion

Multiple sclerosis is characterized by neuroinflammatory, autoimmune, and neurodegenerative processes both directly and indirectly linked to an inappropriate activation of the immune system against CNS “self” antigens. Neuroinflammation is known to directly and systemically contribute to the recruitment of peripheral inflammatory cells and their products within the CNS and to increase BBB permeability. Bidirectional communication between the brain and the peripheral immune system has been well described (36–38), and dysfunction of the BBB, responsible for extensive migration of lymphocytes into the CNS, is considered a hallmark of the MS. The subsequent activation of autoreactive T cells within the CNS, resident microglia and macrophages, would trigger a strong immune response characterized by the secretion of chemokines, cytokines, and growth factors (39) among other events, further causing myelin, axonal, neuronal, and collateral CNS damage (40). The dysfunction of endogenous or exogenous immune cells and their products may be accountable of inflammation-mediated neurodegeneration. The two major endogenous cells in the CNS that drive inflammation are astrocytes and mononuclear phagocytes, which include microglia and perivascular macrophages (41). Cytokines produced in periphery can signal the CNS *via* humoral and neuronal routes. In general, actions occurring either in periphery or within the CNS that are related to immune activation and inflammation display a similar pattern. Understanding the intricate networks of signals pointing out the cross talk between the immune system and CNS in response to different stimuli is one of the main ways of elucidating the pathogenesis of MS and detecting possible targets for more specific therapy. On the basis of these premises, we carried out an observational cross-sectional study in RR-MS patients by simultaneously studying immune and nervous biomarkers that could offer a more integrated view of MS pathophysiology and of IFN-β effects.

Activation of inflammasome directly produces various regulatory or pathogenic actions in cells or tissues such as pyroptosis (42, 43), interference with cellular cytoskeleton arrangement (44), alteration in cell membrane permeability (45), and enhanced lipid metabolism (46), although the induction of classical inflammatory response is the main action. In MS patients, the inflammasome differently regulates cytokine expression. In fact, the level of mRNA expression of genes such as IL-18, IL-1β, IL-1 receptor antagonist, NACHT, LRR, and PYD domain-containing protein 3 (NALP3), and caspase1 is upregulated in PBMCs from RR-MS patients in comparison with healthy donors (47). Studies addressing serum profiles of cytokines that are essential to initiate key immune responses and maintenance of the inflammatory milieu in MS patients are limited and often contradictory. Thus, in this study, we have analyzed different groups of cytokines, adipokines, ACh neurotransmitter, and B-cell differentiation molecules to better understand their networks in naive RR-MS patients compared to IFN-β-treated MS patients and HC.

NACHT, LRR and PYD domain-containing protein 3 inflammasome, besides regulating the release of caspase activation-dependent cytokines IL-1β and IL-18, is also implicated in the production of the HMGB-1. Our data confirm the involvement of pro-inflammatory mediators, such as TNF-α, IL-1β, IL-18, and HMGB-1, matured by NALP3 inflammasome, but also IL-18BP, IL-12/23, in the pathophysiology and clinical course of MS (48). In fact, the capability to activate inflammasome by different adjuvants in experimental MS models drives the occurrence of the disease (49). In addition, our results showed significantly higher serum levels of HMGB-1 in the RR-MS group compared to HC (31%), pointing to a putative role for this inflammatory-like cytokine in RR-MS pathogenesis.

In our RR-MS patients, the mean serum levels of IL-37 were influenced by disease severity. This suggests that IL-37 may be part of a feedback loop to control underlying inflammation in MS pathogenesis. The management of IL-18 signaling requires the presence of its soluble inhibitor IL-18BP, and excessive IL-18BP concentrations may limit the unpleasant effects of the non-mitigated IL-18, either by postponing signaling until appropriate conditions are met or by the eventual degradation or elimination of the protein complex. Several diseases show that an elevation of IL-18BP was in response to increased IL-18. It is interesting to note that a complex of IL-37 and IL-18BP was found to inhibit the production of IL-18-dependent IFN-γ of about 30% more than IL-18BP alone (50). Experimental conditions indicate that IL-37, which signals through IL-18 receptor-α and orphan receptor SIGIRR/IL-1R8 (51), may contribute to IL-18 inhibition. Currently, the *in vivo* impact of IL-37 on IL-18BP inhibition is unknown. Therefore, our results may help to clarify their role in MS disease.

The source of HMGB-1 in MS patients’ serum may be heterogeneous, from injured neurons and astrocytes and from immune-activated cells. By evaluating serum HMGB-1 in MS patients, we observed decreased levels in IFN-β-treated MS patients and a correlation between serum HMGB-1 and indicators of MS disease severity. Our results are consistent with earlier studies showing that higher serum HMGB-1 levels in MS patients may play a pathogenetic role and suggesting that modulation of serum HMGB-1 could be related to the clinical improvement associated with IFN-β in MS patients (52).

Previous studies showed that ACh inhibits IL-1β, IL-18, and HMGB-1 release by suppression of inflammasome activity (53, 54). In a previous study, we have reported that RR-MS patients have lower ACh and higher IL-1β and IL-17 levels in both CSF and serum compared to healthy subjects (53).

Acetylcholine and adipokines behave as hormones participating in neurotransmission and mediation of metabolic processes, respectively, and can modulate innate and adaptive immune cells. The involvement of the cholinergic system in memory and learning functions is clinically evident in neurodegenerative disease. The diminished synthesis of ACh can contribute to memory and cognitive function impairment, these are common clinical manifestations in MS patients. Recent studies have shown the involvement of ACh and adipokines in the pathogenesis of immune-mediated diseases, such as MS (53, 55). A previous study has suggested that inhibition of ACh-esterase (AChE) activity may have a positive effect in the treatment of MS (56, 57), and Mazzanti et al. have shown that IFN-β reduces AChE activity in different brain structures from rats experimentally demyelinated by ethidium bromide (58) improving the hypothesis that ACh modulates the inflammatory process (14).

Our results reinforce the previous observation that a reduction in ACh levels is involved in RR-MS and that ACh and circulating cytokines are mutually influenced (59). Thus, another mechanism for beneficial effect of IFN-β in the modulation of inflammatory cytokines may be the restoration of ACh levels.

Several authors have shown that in MS patients, significantly lower adiponectin and higher leptin correlated with disease disability (EDSS) and may represent a marker of clinical disease activity (60). Inflammatory and immunologic mechanisms in obesity might potentially explain the association between increased risk of MS and adolescent obesity (55, 61). In our cohort of RR-MS patients, we have observed an increased trend of several adipokines, also in relation with EDSS score. Vitamin A plays important roles in immunological responses and brain development, in MS could decrease inflammation and increase tolerance to autoantigens, and may also help in brain protection. RBP-4 is the specific transporter protein of vitamin A, and a correlation between RBP-4 levels and increased BMI was reported. Our cohort of RR-MS patients, all classified as NW subjects, showed higher serum levels of RBP-4 than those in HC and were related to EDSS score.

Visfatin activates NLRP3 inflammasome in endothelial cells *via* ROS-dependent mechanism (62) inducing inter-endothelial junction disruption and is primarily associated with release by endothelial cells. Serum levels of Visfatin-C were higher in our cohort of RR-MS patients than in HC. In contrast, HMGB-1 was significantly downmodulated in MS patients. This ubiquitous nuclear and cytosolic protein is released during sterile inflammation and infection by immune cells, or passively by damaged or necrotic cells, into the extracellular space. Outside the cell, HMGB-1 activates the immune system acting as a chemokine or an alarmin to mediate physiological and pathological responses, such as autoimmunity. Previous studies have demonstrated HMGB-1 can initiate cellular responses primarily by interacting with cell surface receptor for advanced glycation end products and toll-like receptor 4 leading to inflammation, immunity, chemotaxis, and other cell processes (63). On the other hand, adiponectin is an adipocytokine that may contribute to the onset of Alzheimer’s disease (AD) and all-cause dementia (64). Depending on the presence of adiponectin receptors in neurological tissue, the relationship between plasma adiponectin and dementia is still controversial (65–68), and a correlation between serum levels of adiponectin and severity of dementia in NW AD subjects has been reported (69). Several studies reported that upregulation of Visfatin can influence regulation of cytokine secretion and macrophage and neutrophil activation (70). Our data show an increased trend of adipokines’ levels related to EDSS and stress the association between adipocytokines and MS independently from obesity. Thus, we speculate that high levels of adipokines could in turn enhance pro-inflammatory cytokine secretion favoring MS development and chronicity and represent a link among inflammation, autoimmunity, and breakdown of homeostasis. Additional prospective studies with a higher cohort size with MS are required to confirm this hypothesis.

The BAFF/APRIL system is involved in B cell survival, differentiation and class switching, and in size of the peripheral B cell pool. BAFF is the main regulated element of the BAFF/APRIL system, and its serum concentrations were elevated in the majority of our IFN-β-treated MS patients and were associated with more severe clinical disease, whereas the opposite trend was observed for BAFFR. Since B cells can exert both detrimental and beneficial effects, additional evaluation of BAFF/APRIL system is needed.

Understanding the cross talk between the immune and nervous systems and how it can be modulated is necessary for the treatment of neurological diseases such as MS, and our data wedge in the complex mosaic of the mechanisms underlying the control of inflammatory reaction. Taken together, the results from the present study point to the role of several canonical and non-canonical cytokines in MS pathophysiology and course and their variation in response to IFN-β treatment.

## Ethics Statement

The ethics committee of the institutions approved the protocol, and all patients gave their written informed consent for study inclusion, and the study was conducted in accordance with the ethical guidelines of the 2013 Declaration of Helsinki.

## Author Contributions

SS-R, CdeA, and LP recruited and followed patients. MR, CDA, and SS-R designed concept of the research and experiments, analyzed data, and performed critical revision. MR, LP, and EC wrote the draft of the manuscript and performed critical revision. MN and IP designed the study, analyzed data, and prepared figures. SS-R and LF-P acquired data and supervised the work and edited the article.

## Conflict of Interest Statement

The authors declare that the research was conducted in the absence of any commercial or financial relationships that could be construed as a potential conflict of interest.
